# Effect of inhaled nitric oxide on apoptosis of lymphocytes in newborns in a critical state

**DOI:** 10.1186/cc13478

**Published:** 2014-03-17

**Authors:** M Puhtinskaya

**Affiliations:** 1 Research Institute of Obstetrics and Pediatrics, Rostov-on-Djn, Russia

## Introduction

Activation of lymphocyte apoptosis while reducing the endogenous nitric oxide is a predictor of adverse outcome in newborns on mechanical ventilation [1]. With the aim to improve the results of treatment we studied the effect of inhalable nitric oxide on the immune system in newborns with respiratory diseases on mechanical ventilation [2].

## Methods

With the permission of the ethics committee in a controlled, randomized, blind clinical trial we included 27 newborns with respiratory diseases on mechanical ventilation. Randomization was performed by the method of envelopes. Group I (*n *= 17), patients receiving inhalation of nitric oxide at a concentration of 10 ppm for 24 hours controlling the level of methemoglobin (Pulmonox mini; Messer II NO Therapeutics, Austria). Group II (*n *= 10) did not receive inhaled NO. At admission and at 3 to 5 days we studied subpopulations of lymphocytes by one-parameter immunophenotyping using reagents (Immunotech Beckman Coulter, USA): fitz-labeled CD3, CD4, CD8, CD14, CD19, CD34, CD56, CD69, CD71, CD95 monoclonal antibody, the relative content of lymphocytes in early and late apoptosis using Annexin V^+-^labeled FITK and propidium iodide (PL^+^), labeled with PE (Saltag, USA), with results on the Beckman Coulter Epics XL cytometer (USA). The statistical power of the study was 80% (α ≥0.05).

## Results

In Group I relative to group II at 3 to 5 days we registered an increase in mature monocytes (CD14) - 23.1 ± 0.8% (*P *< 0.05); reduction in the relative content of CD69 - 3.8 ± 0.21%, lymphocyte of apoptosis: (Annexin V^-^FITC^+^PI^+^) - 7.12 ± 0.46% and (Annexin V^-^FITC^+^PI^+^) -0. 79.± 0.07% (*P *< 0.001). The duration of mechanical ventilation was 4.1 ± 1.4 days (*P *< 0.05). All patients survived. None of the patients showed clinical or laboratory evidence of adverse effects of inhaled nitric oxide. In Group II seven newborns died, and the duration of mechanical ventilation in survivors was 18 ± 3.4 days.

## Conclusion

Inhalable of nitric oxide activates monocyte-macrophage immunity, stabilizes the apoptosis of T-lymphocytes, and reduces mortality and duration of mechanical ventilation in newborns with respiratory diseases on mechanical ventilation.
